# Mechanistic Studies of the Antiallergic Activity of *Phyllanthus amarus* Schum. & Thonn. and Its Compounds

**DOI:** 10.3390/molecules26030695

**Published:** 2021-01-28

**Authors:** Nur Zahirah Abd Rani, Kok Wai Lam, Juriyati Jalil, Hazni Falina Mohamad, Mohd Shukri Mat Ali, Khairana Husain

**Affiliations:** 1 Drug and Herbal Research Centre, Faculty of Pharmacy, Universiti Kebangsaan Malaysia, Kuala Lumpur 50300, Malaysia; zahira.rani@gmail.com (N.Z.A.R.); david_lam@ukm.edu.my (K.W.L.); juriyatijalil@ukm.edu.my (J.J.); haznifalina@ukm.edu.my (H.F.M.); 2 Horticulture Research Centre, Malaysian Agricultural Research and Development Institute (MARDI), P.O. Box 12301, Kuala Lumpur 50774, Malaysia; mshukri@mardi.gov.my

**Keywords:** *Phyllantus amarus*, antiallergic, RBL-2H3, beta-hexosaminidase, histamine, radioligand, molecular docking

## Abstract

*Phyllanthus amarus* Schum. & Thonn. (Phyllanthaceae) is a medicinal plant that is commonly used to treat diseases such as asthma, diabetes, and anemia. This study aimed to examine the antiallergic activity of *P. amarus* extract and its compounds. The antiallergic activity was determined by measuring the concentration of allergy markers release from rat basophilic leukemia (RBL-2H3) cells with ketotifen fumarate as the positive control. As a result, *P. amarus* did not stabilize mast cell degranulation but exhibited antihistamine activity. The antihistamine activity was evaluated by conducting a competition radioligand binding assay on the histamine 1 receptor (H1R). Four compounds were identified from the high performance liquid chromatography (HPLC) analysis which were phyllanthin (**1**), hypophyllanthin (**2**), niranthin (**3**), and corilagin (**4**). To gain insights into the binding interactions of the most active compound hypophyllanthin (**2**), molecular docking was conducted and found that hypophyllanthin (**2**) exhibited favorable binding in the H1R binding site. In conclusion, *P. amarus* and hypophyllanthin (**2**) could potentially exhibit antiallergic activity by preventing the activation of the H1 receptor.

## 1. Introduction

The Phyllanthaceae family, which was formerly included in the Euphorbiaceae family, is consists of 59 genera, which is considered as a major family in Angiosperm (flowering plants). It is also the plant family for the *Phyllanthus* genus [1,2]. Among the Phyllanthaceae family, the *Phyllanthus* genus, which consists of 884 species, was widely reported for its traditional uses and biological activities. The genus is composed of high contents of lignans, alkaloids, terpenes, flavonoids, tannins, and phenols that are responsible for various bioactivities, such as antiviral, anticancer, antiaging, anti-inflammatory, antioxidant, radioprotective, and hepatoprotective bioactivities [3]. 

Some of the species were reported to have significant antiallergic activity. *Phyllanthus virgatus* G.Forst. leaves extract exhibited antianaphylactic activity through its evaluation using allergic pleurisy, passive cutaneous anaphylaxis, and passive paw anaphylaxis on the rat [4]. Two Ayurvedic formulations containing *Phyllanthus emblica* L. named Chitrak Haritaki Avaleha and Aller-7 were used in the treatment of allergic diseases [5]. Aller-7, a health supplement marketed in the United States of America (USA) consisted of *P. emblica* together with the other six herbs were reported to have antihistaminic, antispasmodic, mast cell stabilization, hyaluronidase inhibition, and lipoxygenase inhibition on allergic rhinitis in vitro model [6]. Aller-7 also significantly reduced three major symptoms of allergic rhinitis, which are nasal congestion, sneezing, and rhinorrhoea upon evaluated clinical trials [7]. 

*Phyllanthus amarus* Schum. & Thonn., which can be found in tropic and subtropic, is traditionally used in combatting various diseases such as but not limited to asthma, fever, diabetes, kidney stones, diuretic, and anemia [8,9]. High interest was given to the plant once it was claimed to have inhibition towards hepatitis B [10], but the research on proving the efficacy of the plant is still ongoing [11,12]. The plant was also evaluated for its potential in treating schistosomiasis [13] and gastric lesion [14]. The plant also exhibited immunomodulatory and immunosuppressive activity [15,16,17] and anti-inflammatory activity [18]. A study observed that the plant possessed an antiasthmatic activity [19,20].

Allergic diseases are a common disease, yet the cases and severity increasing by years. The World Health Organization reported that approximately 20% of the world population suffers from immunoglobulin E (IgE)-mediated allergic diseases. The disease is caused by mast cell degranulation triggered by the presence of allergen. Anti-IgE serum, antihistamines, and mast cell stabilizers are three of the primary treatment for IgE-mediated allergic diseases [21]. The antihistamines and mast cell stabilizers are the most common treatment but caused various side effects such as the dry mouth and sedative effect [22]. Thus, researches are being conducted to find a new antiallergic drug that can cause less side effect on the patient. 

In this study, the antiallergic properties of *P. amarus* and its compounds were determined. The study consisted of its inhibitory activity on allergic reactions biomarkers’ which are beta-hexosaminidase and histamine. Also, the antihistamine activity of the extract and the compounds were examined by identifying its binding energy and binding site on the H1 receptor using radioligand bioassay and molecular docking. 

## 2. Results and Discussion

An allergic reaction is a hypersensitivity of the human immune system towards foreign substances [23]. The reaction is mediated by antibody immunoglobulin E (IgE) [24]. The crosslink between allergen with the high-affinity receptor for immunoglobin E (IgE-FcERI) complex triggers the degranulation of the mast cell, causing the release of vasoactive mediators [25]. The early phase of allergic reaction occurs minutes after the subsequent exposure of an allergen [26]. The crosslinked complex stimulates mast cell degranulation, causing the synthesis of chemokines and cytokines, and the release of lipid-derived mediators and vasoactive amine. These cause allergic reactions such as bronchoconstriction and vasodilation. Two biomarkers in examining allergic reactions are histamine and beta-hexosaminidase [27,28]. Both of these mediators are present in mast cells and basophil, which are released upon mast cell degranulation. Histamine increases the permeability of blood vessels and attracts leukocytes to the allergic site, whereas no specific involvement of beta-hexosaminidase is reported in allergic reactions [27,28]. As both of the markers are constituents of mast cells and basophils granules, the quantification of the biomarkers can determine mast cell degranulation. *P. amarus* is a medicinal plant for various diseases such as diabetes, fever, and kidney stones [8,9]. The plant possesses anti-inflammatory [18], immunomodulatory, and immunosuppressive properties [15,16]. Besides, it was reported that the plant exhibited antiasthmatic activity [19,20]. Thus, the study was conducted to determine whether the plant also exhibited an antiallergic activity. 

### 2.1. HPLC Quantification

**Figure 1:**
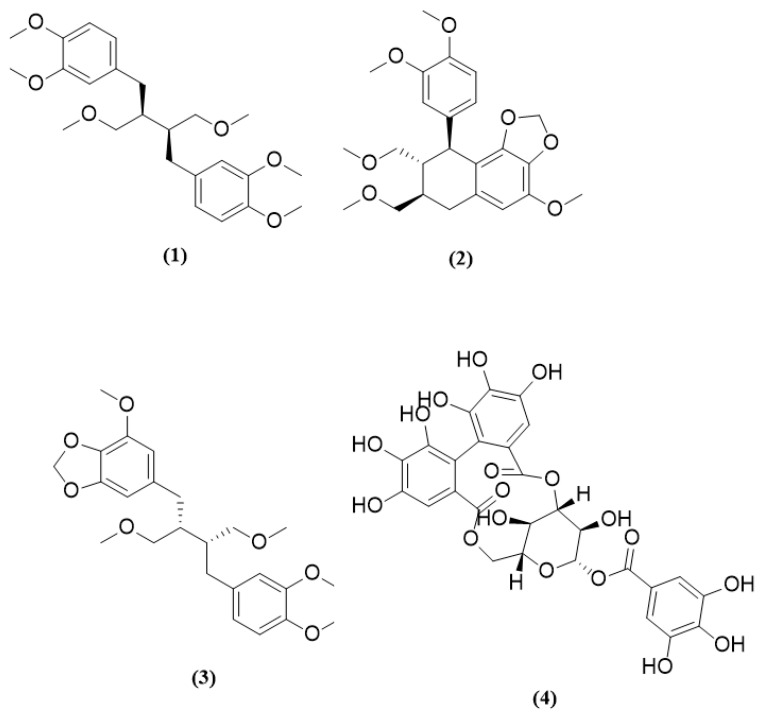
*P. Amarus* compounds: phyllanthin (**1**), hypophyllanthin (**2**), niranthin (**3**), corilagin (**4**).

**Figure 2:**
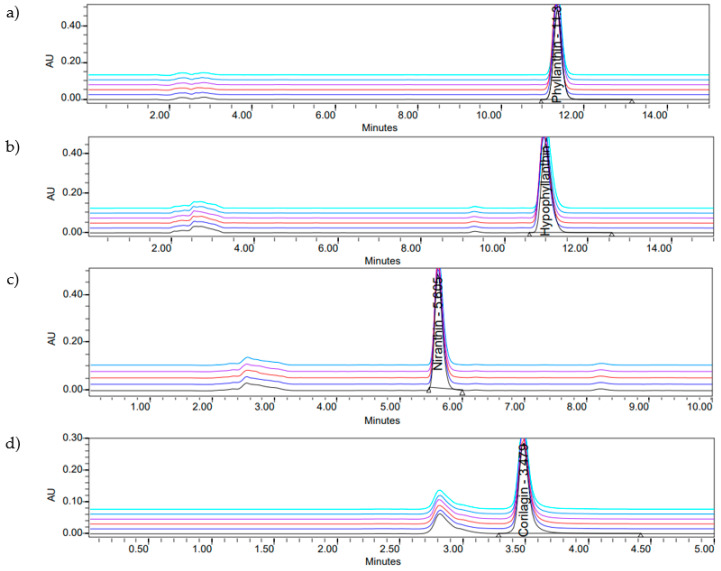
HPLC profile of *P. amarus* compounds: (**a**) phyllanthin; (**b**) hypophyllanthin; (**c**) niranthin; (**d**) corilagin.

**Figure 3:**
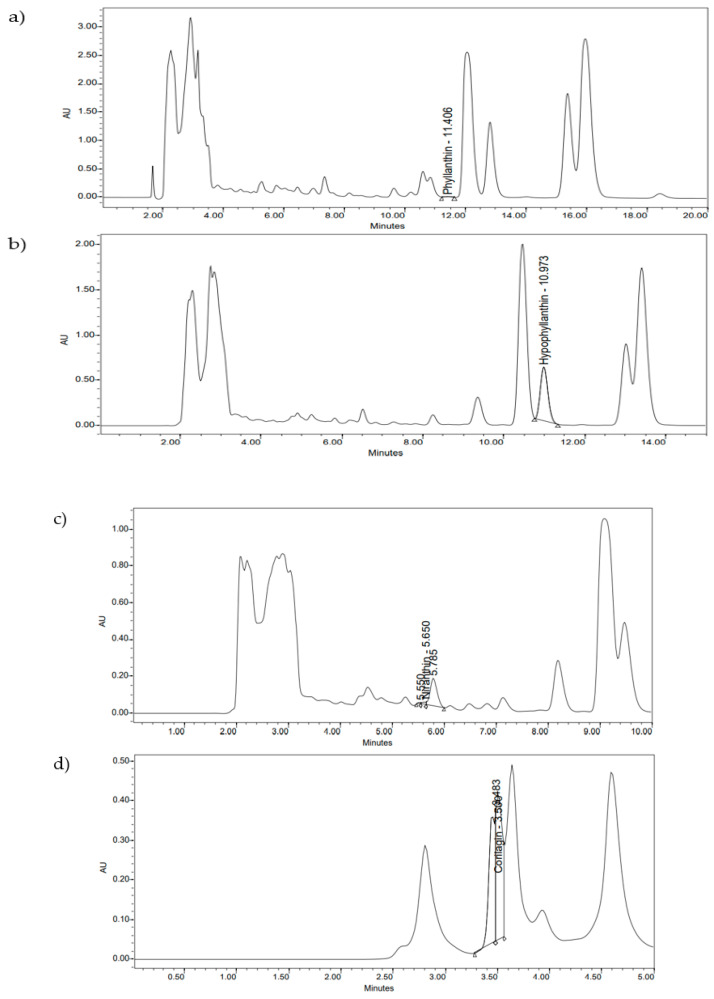
Identification of the compounds in *P. amarus* extract: (**a**) phyllanthin; (**b**) hypophyllanthin; (**c**) niranthin; (**d**) corilagin using HPLC chromatograms.

Phyllanthin (**1**), hypophyllanthin (**2**), niranthin (**3**), and corilagin (**4**) are constituents of *P. amarus* (Figure 1). The crude extract consisted of corilagin (**4**), hypophyllanthin (**2**), niranthin (**3**), and phyllanthin (**1**) from highest to lowest concentration, respectively (Table 1, Table 2, Table 3 and Table 4). The chromatogram of the compounds was documented in Figure 2 and Figure 3.

### 2.2. Cell Viability

**Figure 4:**
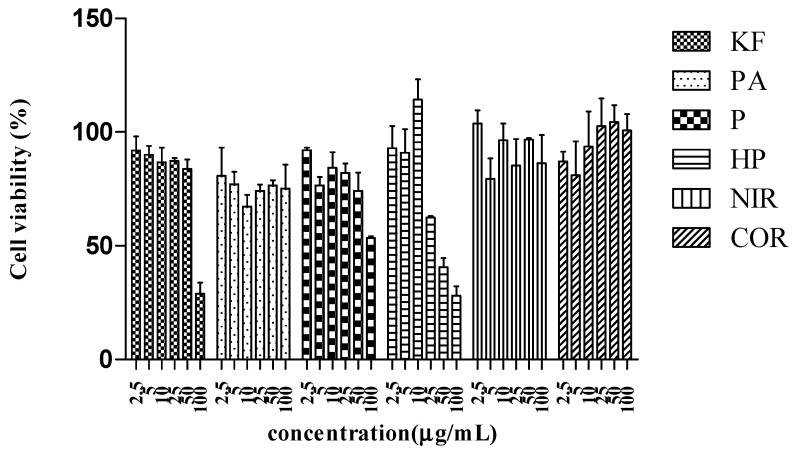
Cell viability of *P. amarus* extract and its compounds towards the RBL-2H3 cell line. Ketotifen fumarate (KF), *P. amarus* (PA), niranthin (NIR), corilagin (COR), phyllanthin (P), hypophyllanthin (HP).

RBL-2H3 cells viability upon different doses of *P. amarus* extract and its compounds was tabulated in Table 5 and Figure 4. Dimethyl sulfoxide (DMSO) at the concentration of 100 µg/mL did not show toxicity to the cell by maintaining more than 70% viability. All concentrations of *P. amarus* extract, niranthin (**3**), and corilagin (**4**) tested were not toxic towards the cell. Meanwhile, only ketotifen fumarate (positive control) and phyllanthin (**1**) concentration at below 50 µg/mL and hypophyllanthin (**2**) concentration at below 25 µg/mL were safe on the cell, maintaining approximately 70% cell viability.

### 2.3. Inhibitory Activity on Beta-Hexosaminidase and Histamine Release from RBL-2H3

**Figure 5:**
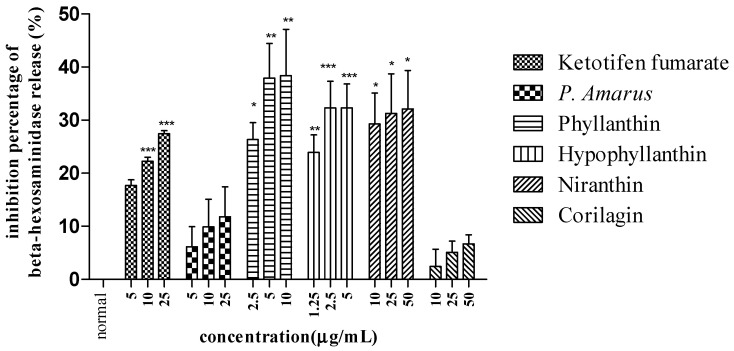
Inhibitory activity of *P. amarus* extract and its compounds towards beta-hexosaminidase release. Data are presented as mean ± SEM (*n* = 3) with significant value of * *p* < 0.05, ** *p* < 0.01, *** *p* < 0.001 as compared to normal.

In examining the effect of *P. amarus* in inhibiting allergic reaction, the concentration of beta-hexosaminidase and histamine upon the pretreatment of *P. amarus* on mast cell degranulation was determined as tabulated in Table 6 and Figure 5.

*P. amarus* extract did not show inhibitory activity on beta-hexosaminidase release. Meanwhile, all of its compounds except corilagin (**4**) showed significant activity in inhibiting beta-hexosaminidase release compared to a normal cell. The compounds exhibited similar activity as ketotifen fumarate, whereas the extract and the compounds did not inhibit histamine release as tabulated in Table 7.

### 2.4. Inhibitory Activity on Beta-Hexosaminidase Activity

**Figure 6:**
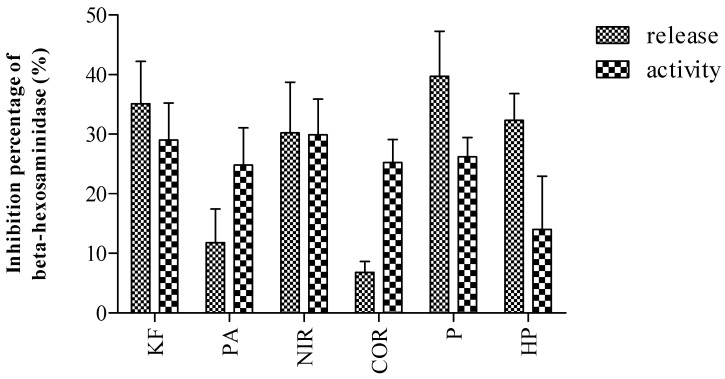
Inhibitory activity of *P. amarus* extract and its compounds towards the beta-hexosaminidase activity. Ketotifen fumarate (KF), *P. amarus* (PA), niranthin (NIR), corilagin (COR), phyllanthin (P), hypophyllanthin (HP).

*P. amarus* did not exhibit mast cell stabilization activity as the extract did not inhibit the release of both the biomarkers; beta-hexosaminidase and histamine. Among the compounds, only the lignans which were phyllanthin (**1**), hypophyllanthin (**2**), and niranthin (**3**) inhibited the release of beta-hexosaminidase. In examining the concentration of beta-hexosaminidase upon mast cell degranulation, one cannot determine whether the concentration was caused by the mast cell stabilizing activity or solely inhibiting the enzyme activity. Thus, in addition to determining the activity of the extract and the compounds on mast cell degranulation, the inhibitory activity of the extract and the compounds directly on the enzyme was also conducted. The inhibitory activity of *P. amarus* extract and its compounds on beta-hexosaminidase activity was tabulated in Figure 6.

It was observed that the compounds inhibited the enzyme activity instead of mast cell degranulation. Based on the result obtained, it was observed that the inhibition was not correlated to its activity on inhibiting mast cell degranulation, as the compounds exhibited similar activity in inhibiting both beta-hexosaminidase release and beta-hexosaminidase activity.

### 2.5. Radioligand Competition Binding Assay

Histamine plays a significant role in exhibiting allergic reactions. The binding of histamine on four types of histamine receptors, which are from the G protein-coupled receptor (GPCR) family, determines the reactions [21]. The H1 receptor stimulates smooth muscle and endothelial cell contraction. The H2 receptor stimulates gastric acid secretion, and the H3 receptor regulates the release of histamine and other neurotransmitters. Meanwhile, the H4 receptor responsible for immune system reaction by regulating cytokine release and mediating the chemotaxis of neutrophils and mast cells. Among the receptors, the receptor that plays a pivotal role in an allergic reaction is the H1 receptor [29]. H1 antagonists, which are also known as an antihistamine, stabilize the H1 receptor by maintaining the receptor state in an inactive form [30]. It will inhibit the release of allergic mediators from mast cells and basophil in addition to inhibiting the chemotaxis of eosinophils and the expression of cell adhesion molecules. There are two types of antihistamine, which are first- and second-generation antihistamine [29]. The first-generation antihistamine penetrates the blood–brain barrier which causes sedative effect while second-generation antihistamine does not.

In determining the affinity of the ligands toward the receptor, competition radioligand binding was conducted. The assay was conducted by measuring the competition binding between a radioligand with a ligand. The concentration of the radioligand with the presence of the ligand and the receptor was determined by using a liquid scintillation counter. It was estimated that upon a prolonged time, the concentration of the radioligand bind to the receptor decreased indicating that the ligand binds to the receptor instead. The Ki value which also known as the binding affinity of the ligand was determined using GraphPad Prism. The low value of Ki indicates that the ligand has a stronger affinity towards the receptor.

From the competition radioligand assay which was maintained at a pH of 7.4 with 1 h incubation time, it was observed that *P. amarus* extract bind to the receptor with a weak affinity (Ki value: 104.2 ng/mL) (Table 8).

In observing specific compounds responsible for the binding activity, it was found out that the compounds showed different strengths of affinity towards the receptor. The highest affinity was showed by hypophyllanthin (**2**) (Ki value: 0.04 nM), followed by niranthin (**3**) (Ki value: 0.44 nM), and lastly phyllanthin (**1**) with the weakest affinity by Ki value of 129.1 nM. In contrast, corilagin (**4**) did not show any affinity towards the receptor. Among the compounds, hypophyllanthin (**2**) with a Ki value of 0.04 nM showed stronger activity than chlorpheniramine with a Ki value of 0.1447 nM. This indicates that hypophyllanthin (**2**) might have potential as an antihistamine drug.

### 2.6. Molecular Docking Studies

**Figure 7:**
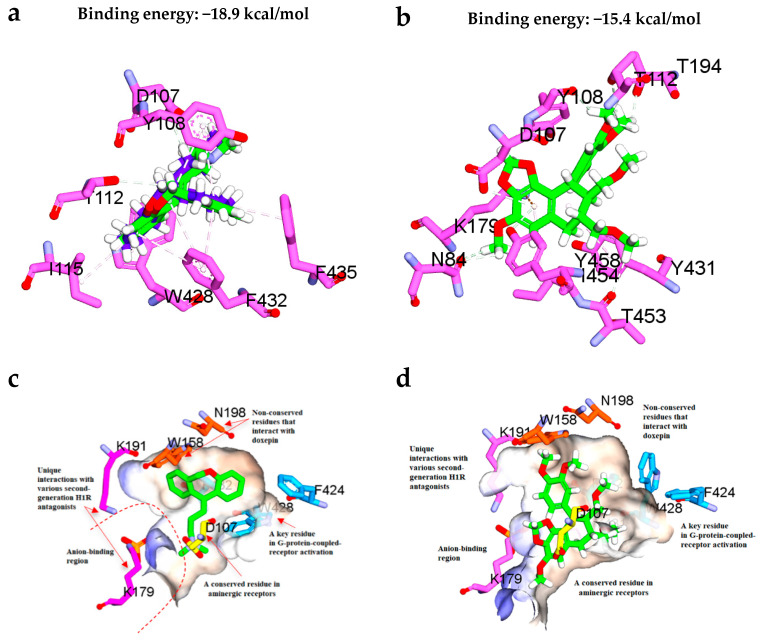
(**a**) Superposition of the docked conformation of doxepin and the original cocrystal doxepin retrieved from the protein crystal structure (PDB ID: 3RZE). The original cocrystal doxepin is colored blue while the docked doxepin is colored green for carbon atoms. (**b**) Binding interactions of the most active compound hypophyllanthin (**2**) with the adjacent residues in the H1 receptor binding site. (**c**) Position of the key residues of the H1 receptor binding site in complex with doxepin as retrieved from the protein crystal structure (PDB ID: 3RZE). (**d**) Position of the key residues of the H1 receptor binding site in complex with hypophyllanthin (**2**) as predicted from the flexible docking.

To check the efficiency of flexible docking protocol implemented in the Discovery Studio 3.1 in reproducing the binding conformation of the cocrystallized ligand doxepin of the Protein Data Bank identifier (PDB ID): 3RZE, the ligand was extracted and then redocked to the binding site. Flexible docking approach was adopted in this study as it allows the side-chains of specified amino acids to move during docking, hence it offers greater realism than other docking formalisms. This would allow the receptor to adapt to different ligands in an induced-fit model. The RMSD between the top ranked pose and the ligand crystal structure was found to be decent with RMSD value of lower than the 2 Å as shown in Figure 7a. Thus, the docking protocol was suitable to be used in this study.

Based on the radioligand binding activity and molecular docking results tabulated in Table 8 and Figure 7, both doxepin and hypophyllanthin were predicted to bind favorably in the H1R binding site with CDOCKER binding energies of −18.9 kcal/mol and −15.4 kcal/mol, respectively. Unlike doxepin, the central core 1,3-benzodioxole ring of hypophyllantin was found to interact with K179 via п-cation interaction, which is commonly observed with the second generation antihistamines as well. Apart from that, the dimethoxy phenyl ring of hypophyllantin forms multiple van der Waals interactions with K191 and the nonconserved W158 and N198 residues. Both K179 and K191 residues are known to form part of the anion-binding region in the H1R. Notably, this region is not conserved, therefore hypophyllantin could possibly exert different selectivity against other aminergic receptors of H1R. This definitely warrants for future study relating to the effects of hypophyllantin on other aminergic receptors. Moreover, it has been reported previously that the presence of a phosphate ion in the anionic region (the phosphate ion is coordinated by K179, K191, Y431 and H450 at the entrance to the ligand-binding pocket could affect the binding of some ligands and the stability of H1R. Thus, the phosphate ion was maintained throughout the docking study. Worth to mention that other important interaction including the van der Waals interaction exerted by the hydrophobic dimethoxy groups of hypophyllantin with the hydrophobic side-chains of F424, W428 and F432. In particular, W428 plays an important role in H1 receptor activation according to Shimamura, et al. [31].

## 3. Materials and Methods

### 3.1. Chemical, Reagents, and Instrument

Ethylenediaminetetraacetic acid (EDTA), sodium chloride (NaCl), potassium chloride (KCl), glucose, magnesium chloride (MgCl_2_), calcium chloride (CaCl_2_), piperazine-*N*,*N*′-bis(2-ethanesulfonic acid) (PIPES), sodium hydroxide (NaOH), citric acid, sodium citrate, p-nitrophenyl-*N*-acetyl-β-D-glucosaminide, sodium carbonate (Na_2_CO_3_), sodium hydrogen carbonate (NaHCO_3_) were obtained from Sigma Aldrich (St. Louis, MO, USA). Phyllanthin, hypophyllanthin, niranthin, and corilagin were purchased from Wuhan Chemfaces BioChemical Co., Ltd. (Wuhan, Hubei, China). 3(4,5-dimethylthiazol-2-yl)-2,5-diphenyltetrazolium bromide (MTT) was obtained from Goldbio Technology (St. Louis, MO, USA).

Ethanol in analysis grade, ortho-phosphoric acid, promethazine hydrochloride, poly(ethyleneimine) solution, and tris, hydrochloride were obtained from Merck KGaa (Darmstadt, Hesse, Germany) and methanol in HPLC grade was obtained from Fischer Scientific (Loughborough, Leicestershire, UK). Acetonitrile and dimethyl sulfoxide (DMSO) were obtained from Merck (Kenilworth, NJ, USA). Minimum essential Eagle media (MEM) with Earle’s salts and L-glutamine, fetal bovine serum (FBS) were obtained from Biowest (Riverside, MO, USA). PBS buffer, pH 7.4 was obtained from Invitrogen (Carlsbad, CA, USA) Trypsin from the beef pancreas and penicillin-streptomycin (10 000 U/mL) were obtained from Gibco, Fischer Scientific (Hampton, NH, USA). Histamine ELISA kit was obtained from Elabscience (Houston, TX, USA).

Pyrilamine (Mepyramine), [pyridinyl 5-3H], Membrane Target Systems: Histamine H1 (human) membrane preparation, in CHO-K1 cells, liquid scintillation cocktail (ULTIMA GOLD MV), pico pro vial, and glass fiber (GF) filter were obtained from Perkin Elmer (Waltham, MA, USA).

Filtermate Harvester (Perkin Elmer, Waltham, MA, USA), Liquid Scintillation Analyzer (Tri-Carb 3110 TR, Perkin Elmer, Waltham, MA, USA), and Quantasmart software were used in the quantification of the radioactive substances in the assay.

### 3.2. Plant Samples and Extractions

*P. amarus* was obtained from Marang, Kuala Terengganu Malaysia with a voucher number of UKMB 30078. The plant was deposited to Herbarium, UKM, Bangi for identification and verification. The whole plant of *P. amarus* (1000.39 g) was dried under room temperature, ground, and macerated using 80% ethanol three times. The crude extract was filtered and evaporated using a rotary evaporator. The crude mass obtained was 11.77 g.

### 3.3. Samples Preparation

In preparation for HPLC analysis, the compounds, phyllanthin (**1**), hypophyllanthin (**2**), niranthin (**3**), and corilagin (**4**) in the quantity of 1 mg were weighed and dissolved in 1 mL of methanol. The compounds were further diluted into 6.25, 12.5, 25, 50, and 100 µg/mL and filtered using a 0.45 µm nylon membrane. The crude extract of *P. amarus* in the concentration of 10 mg was dissolved in 1 mL of methanol and filtered using a 0.45 µm nylon membrane.

In preparation for bioassay analysis, 10 mg of test samples were dissolved in 1 mL of DMSO and were treated as the stock sample. The stock sample was diluted into different concentrations of working test sample 1.25 µM to 50 µM using different solvent (Siraganian buffer, PBS buffer, enriched media) as needed by the bioassays. The working test sample together with its stock sample were kept in 4 °C until further used. Meanwhile, for the radioligand assay, the crude extract was further diluted into concentrations from 10 ng/mL to 0.1 mg/mL while the test compounds were diluted into 300, 100, 10, 1, and 0.1 nM in assay buffer (5 mM MgCl_2_, 50 mM Tris-HCl, pH 7.4). ^3^H mepyramine (50 mM) and promethazine (150 nM/mL) were diluted into working concentrations of 15, 7.5, 3.75, 1.88, 0.94, 0.47, 0.23 and 0.12 nM.

### 3.4. Instrumentation and Chromatographic Condition of HPLC

The HPLC system used was from Breeze with a dual λ absorbance detector (Waters 2487, Massachusetts, USA) isocratic HPLC Pump (Waters 1515 Massachusetts, USA), autosampler (Waters 717 plus Massachusetts, USA). The analysis of phyllanthin (**1**), hypophyllanthin (**2**), niranthin (**3**), and corilagin (**4**) in *P. amarus* extract was obtained from Wu, et al. [31] and Murugaiyah and Chan [32] with modification.

Isocratic mode with a flow rate of 1.0 mL/min and an injection volume of 20 µL using Column Waters X Bridge C18 (4.6 mm × 250 mm, 5 um particle size) was used for the quantification of *P. amarus*. Acetonitrile:water in the ratio of 55:45 was used as a solvent system to quantify phyllanthin (**1**), hypophyllanthin (**2**), and niranthin (**3**) using UV wavelength of 344 nm, whereas, 1% acetic acid in acetonitrile was used as a solvent in quantifying corilagin (**4**) with a UV wavelength of 274 nm.

### 3.5. Analytical Validation for HPLC Quantification

The method for the standards was validated by determining its linearity, the limit of detection (LOD), and limit of quantification (LOQ) as conducted by Jantan, et al. [33]. The linearity was assessed by the calibration curve of the standards of the concentration of 6.25, 12.5, 25, 50, and 100 µg/mL. Two types of precision were conducted which were intra-day precision and interday precision. Intraday precision was determined by injecting six replicates of one of the standard concentrations which were 100 µg/mL. Meanwhile, interday precision was determined by injecting a triplicate of three concentrations of the standards which were 12.5, 25, and 50 µg/mL. The precision was determined by examining the RSD value for the retention time and peak area. Meanwhile, the LOD and LOQ values were calculated based on the slope of the calibration curve using the equation: LOD = 3.3 × standard deviation slope and LOQ = 10 × standard deviation slope.

### 3.6. RBL-2H3 Cells

RBL-2H3 cells were obtained from the Japanese Collection of Research Bioresources (JCRB) cell bank with a cell number of JCRB0023. The cells were cultured in enriched media (MEM containing 10% FBS and 1% penicillin-streptomycin (10 000 U/mL)). Once the cells reaching passage 3 (P3) with 80% confluency and showing stable growth and morphology, the cells were used for the bioassays. Only cells from passage 3 (P3) to passage 5 (P5) were used for the bioassays’ studies.

### 3.7. Cell Viability Assay

The cell viability assay was conducted as reported by Shahari, et al. [34]. RBL-2H3 cells were seeded in 96-well plates in 100 µL at the concentration of 5 × 10^5^ /mL. The cells were incubated overnight for attachment and the medium was removed and supplemented with 100 µL of the test sample in different concentrations (7.81, 15.63, 31.25, 62.5, 125, 250, 500, 1000 µg/mL). The cells were incubated for 4 h. 10 µL of MTT (5 mg/mL in PBS buffer) was added to the mixture and was incubated for 4 h at 37 °C. Upon the appearance of purple formazan crystal, the media was removed and 100 µL of DMSO was added to the well plates to dissolve the crystal. After 10 min of shaking the well plates on a shaker, the absorbance was immediately measured using a microplate reader at 570 nm.

### 3.8. Inhibitory Effects on Beta-Hexosaminidase Release from RBL-2H3

The bioassay was conducted as reported by Abd Rani, et al. [35]. In the concentration of 2 × 10^5^ cells in 400 µL per well, RBL-2H3 cells were seeded and incubated for 2 h. After 2 h, the cells were sensitized with anti-DNP IgE at a concentration of 0.45 µg/mL in 100 µL per well. The cells were incubated overnight until 80% confluent. The cells were washed with 500 µL Siraganian buffer (119 mM of sodium chloride (NaCl), 5 mM of potassium chloride (KCl), 5.6 mM of glucose, 0.4 mM of magnesium chloride (MgCl_2_), 1 mM of calcium chloride (CaCl_2_), 25 mM of piperazine-*N*,*N*′-bis(2-ethanesulfonic acid) (PIPES), 40 mM of sodium hydroxide (NaOH), 0.1% BSA; pH 7.2) twice before were added with 160 µL of Siraganian buffer and incubated for 10 min. The cells were then treated with different concentrations of test samples in 20 µL. After 10 min of incubation, allergen, DNP-BSA at a concentration of 10 mg/mL were added to the cells. The cells were incubated for another 30 min. The supernatant (50 µL) was transferred into 96-well plates and was added immediately with 50 µL of the substrate (1 µM *p*-nitrophenyl-*N*-acetyl-β-D-glucosaminide in 0.1 M citrate buffer, pH 4.5). The cells were further incubated for 1 h and the reaction was stopped using a 200 µL stop solution (0.1 M Na_2_CO_3_/NaHCO_3_, pH 10.0). The absorbance was measured using the microplate reader at 405 nm. The percentage of inhibition was measured using Equation (1). Control cell was assumed to have 100% beta-hexosaminidase release with allergen added without any treatment while the normal cell was assumed to have 0% beta-hexosaminidase release with cell without any allergen and treatment added to the well. The blank well consisted of the test sample. Ketotifen fumarate was used as a positive control.

(1) $$\mathrm{Percentage}\;\mathrm{inhibition}\;\left(\%\right)\;=\;(1-\;\frac{\mathrm{OD}\;\mathrm{test}\;\mathrm{sample}-\mathrm{OD}\;\mathrm{blank}-\mathrm{OD}\;\mathrm{normal}}{\mathrm{OD}\;\mathrm{control}-\mathrm{OD}\;\mathrm{normal}})\;\times \;100$$

### 3.9. Inhibitory Activity on Beta-Hexosaminidase Activity

The bioassay was conducted as reported by Abd Rani, et al. [35]. RBL-2H3 cells (5 × 10^6^ cells) in 10 mL of PBS buffer were sonicated for 20 min. The cells were then centrifuged at 1500 rpm for 5 min before being used for the assay. Forty-five microliters of the supernatant was transferred into a 96-well plate and treated with 5 µL of the highest viable concentration of the test samples or ketotifen fumarate. 50 µL of the substrate (1 mM *p*-nitrophenyl-*N*-acetyl-β-D-glucosaminide in 0.1 M citrate buffer, pH 4.5) was added immediately to the mixture and was incubated for 1 h at 37 °C. The reaction was stopped by adding 200 µL of stop solution (0.1 M Na_2_CO_3_/NaHCO_3_, pH 10.0) and the absorbance was measured using a microplate reader at 405 nm. The percentage of inhibition was measured using equation [1]. The control well was assumed to have 100% beta-hexosaminidase enzyme without any treatment while the normal cell was PBS buffer. The blank well consisted of the test sample in PBS buffer.

### 3.10. Inhibitory Effects on Histamine Release from RBL-2H3

The bioassay was conducted as reported by Shahari, et al. [36] and Abd Rani, et al. [35]. In 24-well plates, 2 × 10^5^ of RBL-2H3 cells were seeded in 400 µL per well. The cells were sensitized with 0.45 µg/mL anti-DNP IgE in 100 µL after two hours of incubation. The cells were incubated overnight until reaching 80% confluency. The cells were washed twice with 500 µL of enriched media before being constituted with 320 µL enriched media. After 10 min of incubation, the cells were treated with 40 µL of different concentration of test compounds and was incubated for another 10 min. The cells were treated with 20 µL of allergen, DNP-BSA (10 mg/mL), and further being incubated for another 30 min. The supernatant obtained was transferred into a microcentrifuge tube, and centrifuged for 20 min at 1000× *g* at 8 °C. The supernatant was transferred to the Histamine ELISA kit and the histamine quantification was conducted based on the ELISA manufacturer protocol given. The percentage of inhibition was measured using Equation (2). The control cell was assumed to have 100% histamine release with allergen added without any treatment while blank well corresponded to 0% cytokine release. Ketotifen fumarate was used as a positive control.

(2) $$\mathrm{Percentage}\;\mathrm{inhibition}\;\left(\%\right)\;=\;(1-\;\frac{\mathrm{OD}\;\mathrm{test}\;\mathrm{sample}-\mathrm{OD}\;\mathrm{blank}}{\mathrm{OD}\;\mathrm{control}-\mathrm{OD}\;\mathrm{blank}})\;\times \;100$$

### 3.11. Radioligand Competition Binding Assay

The radioligand assay was conducted as reported by Strakhova and Esbenshade [37]. In 96 well plates, 25 µL of 1.5 nM 3H-mepyramine was added with different concentrations of the test samples. The mixture was immediately added with 200 µL of histamine receptor membrane (5 µg protein/well). The mixture was incubated for an hour at room temperature while shaking on the shaker. After an hour incubation, the mixture was harvested on GF/B uni filter presoaked with 0.5% PEI for 3 min. The GF/B uni filter was rinsed a few times with a wash buffer before being dried for one and a half hours. Once the uni filter was dry, the punched filter was transferred to 4 mL pico pro vial and was soaked with 2 mL of liquid scintillation cocktail. The solution was vortexed for a few seconds before being quantified using the liquid scintillator. Ki value (nmol/L) was obtained by using Equation (3). Chlorpheniramine was used as a positive control.

(3) $$\mathrm{Ki}\;\mathrm{value}\;(\mathrm{nmol}/L)\;=\;\frac{\mathrm{IC}\;50}{1+2.75\;\mathrm{Kd}}$$

### 3.12. Molecular Docking

To elucidate the possible binding interactions of the most active compound hypophyllanthin (**2**) with the H1 receptor, the protein crystal structure (PDB ID: 3RZE with resolution 3.1 Å) was retrieved from Protein Data Bank [31]. All existing cocrystallized water molecules were removed prior to the docking experiment (*located far away from the binding site). A phosphate ion located in the anion-binding region at the entrance to the ligand-binding pocket was included in the study as it has been shown to affect the stability of the receptor and binding of some of the ligands [31]. The 2D and 3D structures of the cocrystallized ligand, doxepin and hypophyllanthin (**2**) were drawn in ChemDraw Ultra 12.0 and were minimized in Discovery Studio 3.1 (Accelrys, San Diego, CA, USA). The following residues were set as flexible during the docking process: V76, V80, L104, S105, M106, D107, Y108, V109, S111, T112, S114, I115, W158, I162, K179, T182, F184, K191, T194, N198, F199, Y200, F424, W428, Y431, F432, F435, T453, I454 and Y458. Flexible docking was performed according to the standard protocol implemented in the Discovery Studio^®^ 3.1 (Accelrys, San Diego, CA, USA) [38,39,40]. The final docking parameters included: (1) the “number of hotspots” was set to 100; (2) the docking tolerance set to 0.25; (3) the “conformation method” was set to “BEST” and the other parameters were kept at the default settings. The following steps were performed: (i) receptor conformations were calculated using ChiFlex; (ii) ligand conformations were created; (iii) ligand docking into active protein conformation site was performed using LibDock; (iv) similar ligand poses were removed by clustering (poses were clustered regardless of the protein conformation since the protein conformations were rebuild during the next step); (v) selected protein side-chains were refined in the presence of the rigid ligand using ChiRotor and (vi) final ligand refinement was performed using CDOCKER. Finally, to validate the docking protocol, the cocrystallized ligand, doxepin was removed and redocked to the binding site using the same method described above.

### 3.13. Statistical Analysis

All of the bioassays were conducted as triplicate (*n* = 3) and were reported as the mean value ± standard error of the mean (SEM). The data were statistically analyzed using GraphPad Prism 5 to obtain Kd value (nmol/L), Ki value (nmol/L), and IC_50_ value with 95% confidence intervals. The data were statistically analyzed using GraphPad Prism 5 using one-way ANOVA with a *p*-value of *p* < 0.05 to be claimed as significant.

## 4. Conclusions

The study concludes that *P. amarus* did not inhibit mast cell degranulation but exhibited a weak antihistamine activity by binding to the H1 receptor. Four compounds of *P. amarus* were quantified with the highest amount of corilagin (**4**), followed by hypophyllanthin (**2**), niranthin (**3**), and phyllanthin (1), respectively. Similar to the extract, the compounds did not possess mast cell stabilization activity as they only inhibited the activity of the beta-hexosaminidase enzyme, not mast cell degranulation. Based on the in vitro experiment and molecular docking results on H1R receptor, the most active compound hypophyllanthin (**2**) could possibly exert its antihistamine activity by binding with the H1 receptor and interfering with its activation.

## Figures and Tables

**Table 1:** Linearity, limit of detection (LOD) and limit of quantification (LOQ) of compounds.

Chemical Marker	Concentration Range (µg/mL)	Linear Equation	R-Squared Value	LOD (µg/mL)	LOQ (µg/mL)
Phyllanthin (**1**)	6.25–100	y = 65,900x + 138,000	0.999747	3.514099	10.64879
Hypophyllanthin (**2**)	6.25–100	y = 73,400x + 484,000	0.984817	13.52513	40.98525
Niranthin (**3**)	6.25–100	y = 41,900x + 201,000	0.996362	5.452889	16.52391
Corilagin (**4**)	6.25–100	y = 16,100x + 983,000	0.982418	14.46484	43.83286

**Table 2:** Intra-assay precision of compounds.

Chemical Marker	Concentration (µg/mL)	RSD% Retention Time	RSD% Peak Area
Phyllanthin (**1**)	100	0.121	0.15
Hypophyllanthin (**2**)	100	0.175	0.39
Niranthin (**3**)	100	0.057	0.63
Corilagin (**4**)	100	0.052	0.91

RSD: relative standard deviation.

**Table 3:** Intermediate precision of compounds.

Chemical Marker	Concentration (µg/mL)	RSD% Retention Time	RSD% Peak Area
Phyllanthin (**1**)	12.5	0.12	6.30
	25	0.17	2.34
	50	0.20	1.34
Hypophyllanthin (**2**)	12.5	0.67	8.66
	25	0.36	8.36
	50	0.31	9.39
Niranthin (**3**)	12.5	0.69	6.19
	25	0.39	8.95
	50	0.19	6.70
Corilagin (**4**)	12.5	0.32	7.86
	25	0.40	18.77
	50	0.40	6.89

RSD: relative standard deviation.

**Table 4:** Quantification of *P. amarus* compounds in the crude extract.

Chemical Marker	Concentration (µg/mL)
Phyllanthin (**1**)	0.22
Hypophyllanthin (**2**)	98.24
Niranthin (**3**)	1.89
Corilagin (**4**)	90.24

**Table 5:** Cell viability of *P. amarus* and its compounds towards the RBL-2H3 cell line.

Concentration	2.5 µg/mL	5 µg/mL	10 µg/mL	25 µg/mL	50 µg/mL	100 µg/mL
Ketotifen fumarate (positive control)	91.93 ± 6.16	90.09 ± 3.89	86.75 ± 6.47	87.37 ± 1.17	83.84 ± 4.10	28.89 ± 4.87
*P. amarus*	80.81 ± 12.36	77.04 ± 5.55	67.25 ± 5.20	74.22 ± 2.69	76.59 ± 2.23	75.18 ± 10.50
Phyllanthin (**1**)	92.02 ± 1.13	76.57 ± 3.77	84.30 ± 6.83	82.10 ± 4.03	74.18 ± 7.99	53.48 ± 0.65
Hypophyllanthin (**2**)	92.96 ± 9.69	90.95 ± 10.40	114.35 ± 8.89	62.46 ± 0.56	40.58 ± 4.17	28.12 ± 4.13
Niranthin (**3**)	103.79 ± 5.79	79.44 ± 8.96	96.46 ± 7.33	85.37 ± 11.62	96.63 ± 0.63	86.26 ± 12.41
Corilagin (**4**)	87.10 ± 4.31	81.10 ± 14.81	93.71 ± 15.36	102.69 ± 12.10	104.43 ± 7.35	100.75 ± 7.2
DMSO (solvent)	83.25 ± 14.49	82.12 ± 8.73	88.94 ± 9.52	97.73 ± 6.65	83.74 ± 14.92	74.56 ± 15.31

**Table 6:** Inhibitory activity towards the beta-hexosaminidase release of *P. amarus* extract and its compounds.

Concentration	1.25 µg/mL	2.5 µg/mL	5 µg/mL	10 µg/mL	25 µg/mL	50 µg/mL
Ketotifen fumarate (positive control)	-	-	17.71 ± 1.05	22.23 ± 0.75 ***	27.43 ± 0.58 ***	-
*P. amarus*	-	-	6.14 ± 3.82	9.88 ± 5.22	11.80 ± 5.62	-
Phyllanthin (**1**)	-	26.35 ± 3.18 *	37.93 ± 6.51 **	38.40 ± 8.72 **	-	-
Hypophyllanthin (**2**)	23.94 ± 3.31 **	32.33 ± 4.99 ***	32.32 ± 4.51 ***	-	-	-
Niranthin (**3**)	-	-	-	29.29 ± 5.81 *	31.28 ± 7.43 *	32.09 ± 7.27 *
Corilagin (**4**)	-	-	-	2.43 ± 3.26	5.12 ± 2.09	6.68 ± 1.71

Data are presented as mean ± standard error of mean (SEM) (*n* = 3) with significant value of * *p* < 0.05, ** *p* < 0.01, *** *p* < 0.001 as compared to normal.

**Table 7:** Inhibitory activity towards histamine release of *P. amarus* extract and its compounds.

Concentration	1.25 µg/mL	2.5 µg/mL	5 µg/mL	10 µg/mL	25 µg/mL
Ketotifen fumarate (positive control)	-	-	3.90 ± 6.80	44.74 ± 23.01	58.74 ± 23.16
*P. amarus*	-	40.26 ± 27.12	11.22 ± 28.00	−6.99 ± 36.16	-
Phyllanthin (**1**)	-	−3.05 ± 7.93	55.80 ± 19.31	−11.64 ± 70.80	-
Hypophyllanthin (**2**)	16.39 ± 46.64	11.89 ± 64.33	71.20 ± 34.68	-	-
Niranthin (**3**)	-	-	34.74 ± 12.17	58.96 ± 22.24	41.22 ± 31.24
Corilagin (**4**)	nd	nd	nd	nd	nd

Data are presented as mean ± SEM (*n* = 3). nd: not defined.

**Table 8:** Radioligand binding activity of *P. amarus* extract and its compounds.

Ligands	Radioassay (Ki) (nM)
Doxepin (positive control)	-
Chlorpheniramine (positive control)	0.1447
*P. amarus*	104.2 ng/mL
Phyllanthin (**1**)	129.1
Hypophyllanthin (**2**)	0.04
Niranthin (**3**)	0.4436
Corilagin (**4**)	Not converged

## Data Availability

The data presented in this study are available on request from the corresponding author.

## Sample Availability

Samples of the compounds are not available from the authors.
